# Evidence-based diagnostic use of VEMPs: From neurophysiological principles to clinical application

**DOI:** 10.1007/s00106-019-00767-2

**Published:** 2020-06-12

**Authors:** J. Dlugaiczyk

**Affiliations:** Deutsches Schwindel- und Gleichgewichtszentrum (DSGZ), Klinikum der Universität München, LMU München, Marchioninistraße 15, 81377 Munich, Germany

**Keywords:** Vestibular evoked myogenic potentials, Vibration, Otolithic membrane, Bone conduction, Vestibular neuritis

## Abstract

**Background:**

Vestibular evoked myogenic potentials (VEMPs) are increasingly being used for testing otolith organ function.

**Objective:**

This article provides an overview of the anatomical, biomechanical and neurophysiological principles underlying the evidence-based clinical application of ocular and cervical VEMPs (oVEMPs and cVEMPs).

**Material and methods:**

Systematic literature search in PubMed until April 2019.

**Results:**

Sound and vibration at a frequency of 500 Hz represent selective vestibular stimuli for the otolith organs. The predominant specificity of oVEMPs for contralateral utricular function and of cVEMPs for ipsilateral saccular function is defined by the different central projections of utricular and saccular afferents. VEMPs are particularly useful in the diagnosis of superior canal dehiscence and otolith organ specific vestibular dysfunction and as an alternative diagnostic approach in situations when video oculography is not possible or useful.

**Conclusion:**

The use of VEMPs is a simple, safe, reliable and selective test of dynamic function of otolith organs.

**Electronic supplementary material:**

The online version of this article (10.1007/s00106-019-00767-2) contains supplementary material with further information on the following topics: S1: The striola: an ideal “jerk” detector. S2: Sound and vibration as otolithic stimuli. S3: Vestibular microphonics. S4: Superior canal dehiscence. Contribution and additional material are available at www.springermedizin.de. Please enter the title of the article in the Search, the supplementary material can be found at the end of the article under “Additional content”.

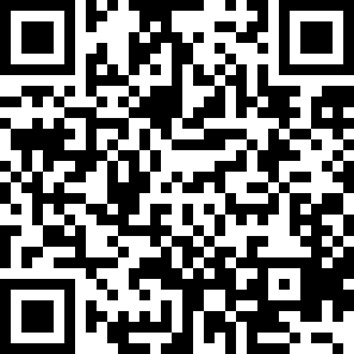

## Introduction

Vestibular evoked myogenic potentials (VEMPs) are short-latency, otolith-driven reflexes elicited by air-conducted sound (ACS), bone-conducted vibration (BCV), or galvanic vestibular stimulation and recorded from ocular (oVEMPs) or cervical (cVEMPs) muscles [5, 71, 74]. Combining VEMPs with the video head impulse test (vHIT) [36] allows for a receptor-specific examination of all five vestibular endorgans in clinical practice (oVEMPs: utricle; cVEMPs: saccule; vHIT: semicircular canals; [14]).

## Reflex pathways

**Fig. 1:**
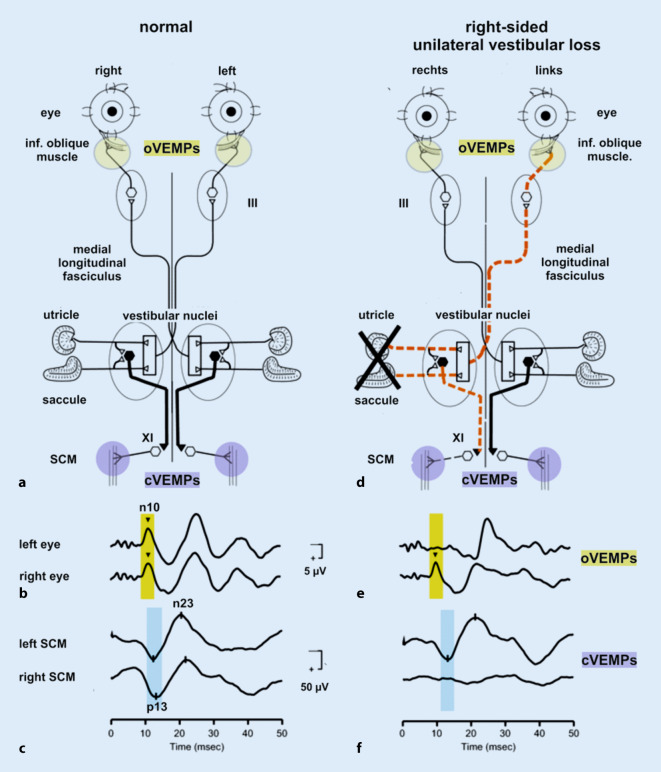
Reflex pathways and VEMPs recorded in **a–c** a healthy subject and **d–f** a patient with right-sided unilateral vestibular loss (uVL). *III* oculomotor nucleus; *XI* spinal accessory nucleus; *black hexagon* inhibitory interneurons in the vestibular nuclei projecting to the motoneurons of the ipsilateral sternocleidomastoid muscle (*SCM*). **a–c** In a healthy subject, symmetric oVEMP n10 responses are recorded from the inferior oblique muscle beneath the left and right eyes. Likewise, symmetric cVEMP p13n23 responses are present in the left and right SCM. **d–f** In a patient with right-sided uVL (X), however, contralateral (= *left*) oVEMPs (crossed reflex pathway) and ipsilateral (= *right*) cVEMPs (uncrossed reflex pathway) are absent. VEMPs vestibular evoked myogenic potentials, *c* cervical, *o* ocular, *inf* inferior. (Slightly modified and reprinted from [17] with permission from © John Wiley & Sons)

Ocular VEMPs (oVEMPs) are mediated by a predominantly crossed reflex pathway projecting from the utricle to the ipsilateral vestibular nuclei and *via* the medial longitudinal fasciculus to the contralateral oculomotor nucleus (N. III) that supplies the contralateral inferior oblique muscle (Fig. 1a). From there, an early excitatory potential (n10) is recorded by surface electrodes approximately 10 ms after stimulus onset while the subject is looking upwards (Fig. 1b; [17, 25]).

The oVEMP n10 amplitude predominantly reflects contralateral utricular function.

The oVEMP n10 amplitude predominantly reflects contralateral utricular function

In contrast, cVEMPs involve a mainly uncrossed reflex circuit running from the saccule to the ipsilateral vestibular nuclei from where inhibitory interneurons project to the ipsilateral spinal accessory nucleus (N. XI) that innervates the sternocleidomastoid muscle (SCM). The early inhibitory potential (p13n23) recorded from the activated SCM between 13 and 23 ms after stimulus onset is an indicator of predominantly ipsilateral saccular function (Fig. 1c; [17]).

The cVEMP p13n23 amplitude is predominantly an indicator of ipsilateral saccular function.

The cVEMP p13n23 amplitude is predominantly an indicator of ipsilateral saccular function

VEMPs are vestibular—and not cochlear—reflexes. They are preserved in subjects with profound sensorineural hearing loss, but intact peripheral vestibular function. On the other hand, they are absent in patients after vestibular neurectomy with normal hearing [5, 41–43, 71]. While sensorineural hearing loss does not affect VEMPs, it should however be noted that a conductive hearing loss (air–bone gap) as small as 10 dB may cancel ACS-evoked VEMP responses completely ([86]; for details see the section “Nature of the stimulus”).

## Relevant anatomy and physiology of the otolith organs

**Fig. 2:**
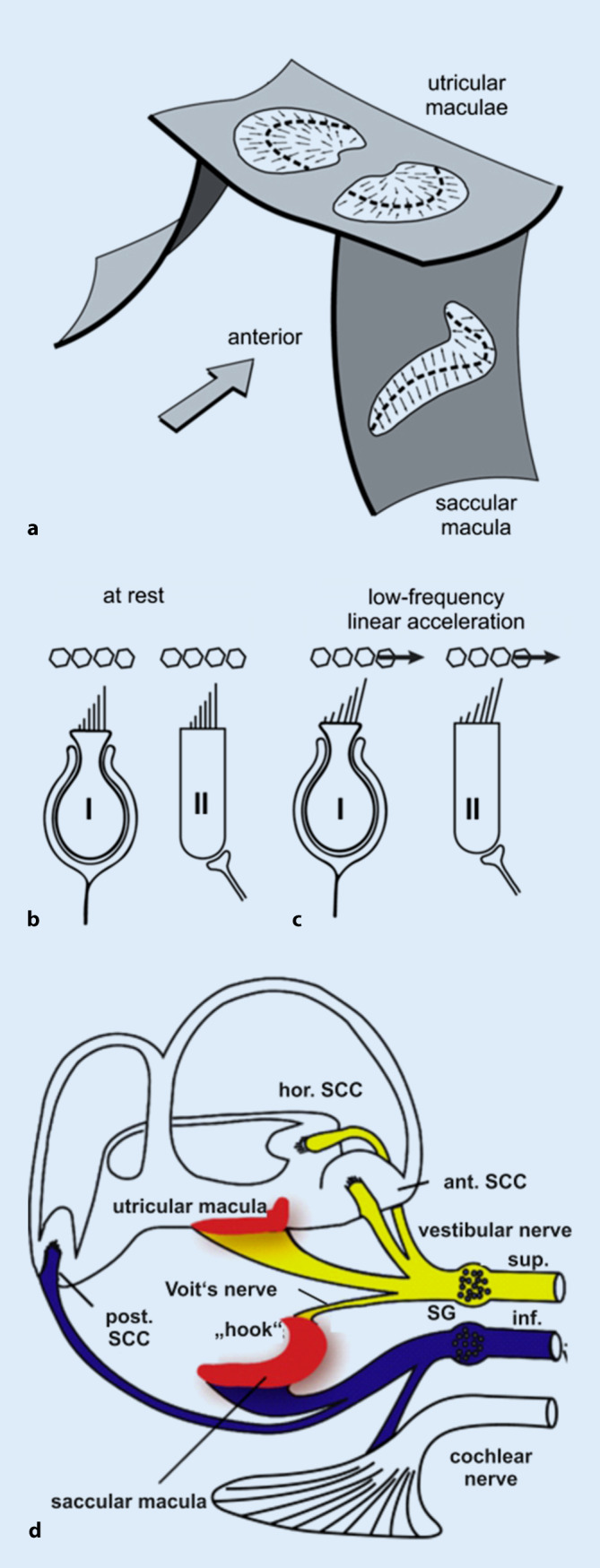
Schematic diagrams of **a** the otolith organs, **b,c** their vestibular hair cells and **d** their afferent innervation. **a** Spatial orientation of the utricular and saccular maculae in the labyrinth. *Dashed lines:* lines of polarity reversal/striola. *Arrows:* polarization vectors of the vestibular hair cells. For details see [69]. **b** Amphora-shaped type I vestibular hair cell (with calyx synapse) and cylindrical type II vestibular hair cell (with bouton synapse) and the overlying otoconia at rest: no deflection of hair cell stereocilia. **c** Vestibular hair cells and otoconia during constant or low-frequency linear acceleration: relative motion of the otoconial membrane relative to the neuroepithelial layer and deflection of hair cell stereocilia opposite to the direction of linear acceleration. (Slightly modified and reprinted from [21] with permission from © I.S. Curthoys et al., CC BY 4.0 [https://creativecommons.org/licenses/by/4.0/]). **d** Afferent innervation of the vestibular organ. *Yellow*: superior vestibular nerve; *blue*: inferior vestibular nerve; *SG* Scarpa’s ganglion; *ant.*/*hor.*/*post. SCC* anterior (= superior)/horizontal/posterior semicircular canal. (Reprinted from [17] with permission from © John Wiley & Sons)

**Fig. 3:**
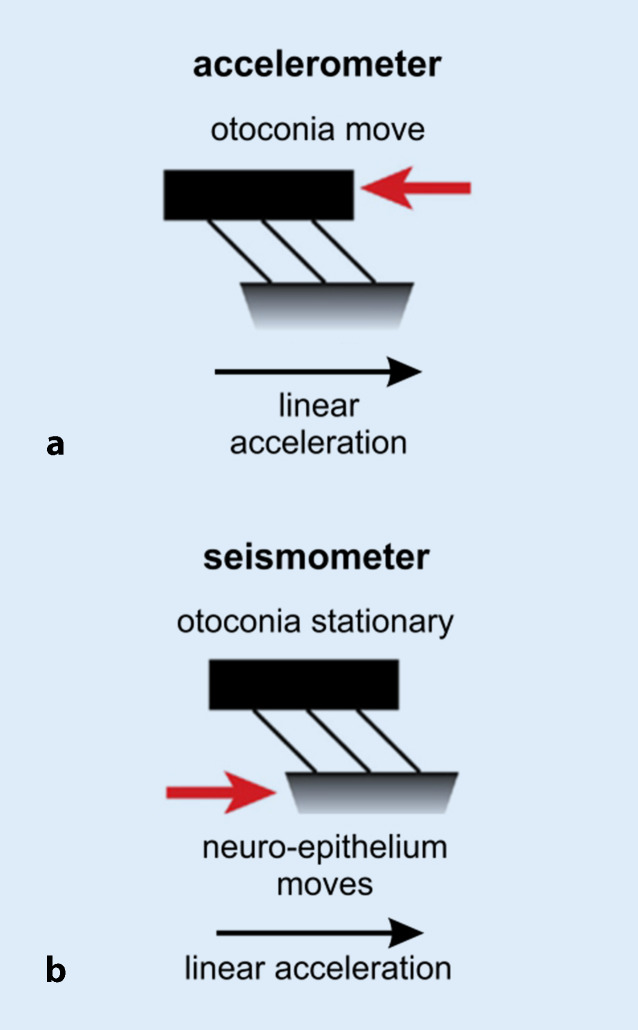
Dual mode of action of the otolith organs as **a** accelerometers for constant and low-frequency linear accelerations (= sustained channel of otolithic function) and **b** seismometers for high-frequency changes in acceleration (= transient channel). (Reprinted from [21] with permission from © I.S. Curthoys et al., CC BY 4.0, https://creativecommons.org/licenses/by/4.0)

The otolith (macula) organs sense linear acceleration, the utricle predominantly in the horizontal plane and the saccule predominantly in the vertical plane (Fig. 2a; [58, 69]). During constant or low-frequency linear acceleration, the otolith organs work as accelerometers, i.e., the otoconial membrane lags behind the underlying neuro-epithelium due to the inertia of the otoconia, thus, causing a relative movement between the two layers that is opposite to the direction of linear acceleration (Figs. 2b, c and 3a; [21, 34]). The resulting shearing force deflects the hair cell bundles of vestibular hair cells in the neuro-epithelial layer. Deflection of the stereocilia towards the kinocilium results in depolarization of the hair cell, thus, triggering the signal transduction process between the vestibular hair cell and its postsynaptic afferent nerve fiber.

Otolith afferents are organized in two different functional channels [29, 31]. The so-called transient (= dynamic) system, which is particularly sensitive to changes in linear acceleration (“jerks”), receives its input from highly specialized type I striolar vestibular hair cells that are connected to postsynaptic vestibular afferents with irregular resting activity *via* ultrafast calyx synapses (Fig. 2b, c; for more details see S1 [online supplementary material] and [9, 76]). On the other hand, constant and low-frequency linear accelerations are processed by the sustained (= transient) channel that involves mainly type II vestibular hair cells and regular vestibular afferents from the extrastriola [20, 21, 23, 26].

While all utricular afferents run in the superior vestibular nerve, 90% of saccular afferents are organized in the inferior vestibular nerve and 10% in the superior vestibular nerve. The so-called “Voit’s nerve” contains mainly afferents from the hook region of the saccular macula (Fig. 2d; [24]).

## Sound and vibration as otolithic stimuli

In clinical practice, usually sound and vibration of 500 Hz and above are applied to evoke VEMPs. How can these—with regard to the vestibular system—high frequencies provide adequate linear acceleration for the otolith organs, a seemingly sluggish system whose function is based on the inertia of the otoconia?

### Biomechanical evidence

Each cycle of a sound wave (ACS or BCV) is able to create a pressure wave—and thus a change in acceleration—in the endolymph surrounding the otolith maculae (for details see S2 [online supplementary material]). Accordingly, a stimulus frequency of 500 Hz is equivalent to 500 changes in acceleration per second, making it an ideal “jerk” stimulus for the transient vestibular system [21]. The conventional “accelerometer” mode of the otolith organs (Fig. 3a) would, however, be too sluggish to encode this high-frequency acceleration with adequate temporal precision. Instead, the utricle and the saccule work as seismometers in this situation—similar to the mechanism by which technical seismometers detect earthquakes (see Infobox 1 for further information about the mode of action of a seismometer): the neuro-epithelium oscillates with the stimulus frequency, while the otoconia remain stationary because of their inertia (Fig. 3b; [34]) resulting in a relative motion between the otoconial membrane and the neuro-epithelium, albeit with reversed roles of the two layers as compared to the accelerometer mode. Finally, this relative motion causes deflection of the hair cell stereocilia and, thus, hair cell depolarization (see above and [21]).

Experimental evidence for the “seismometer” mode of otolith organs derives from a sophisticated guinea pig model showing that application of sound or vibration to the guinea pig’s skull resulted in periodic oscillations of the utricular macula and de-/repolarizations of the utricular hair cells in sync with the stimulus frequency up to several kHz (for details see S3 [online supplementary material] and [65, 66]).

It is the dual mode of action as accelerometers and seismometers that allows the otolith organs to encode both constant/low-frequency linear acceleration and high-frequency sound and vibration. The 500 Hz ACS or BCV stimulus applied in clinical VEMP testing is ideally suited to activate the transient (= dynamic) otolithic system [21].

### Neurophysiological evidence

How does sound- and vibration-induced depolarization of vestibular hair cells activate vestibular afferents? And which types of afferents: otolith or semicircular canal? Those with irregular or regular resting discharge? The answers to these questions were mainly found by performing extracellular recordings of primary vestibular neurons in Scarpa’s ganglion (SG) during application of ACS or BCV to a guinea pig’s skull (Fig. 2d). The measurement set-up, in particular the stimulus parameters, were chosen to match the conditions during VEMP testing in humans [10, 13, 15, 18, 21, 22].

**Fig. 4:**
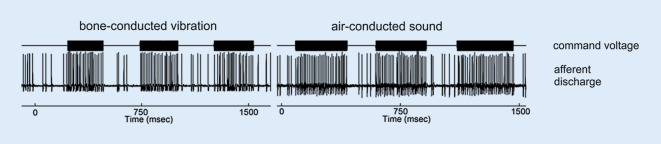
Response of an utricular afferent neuron with irregular resting discharge to 500 Hz bone-conducted vibration (BCV) and air-conducted sound (ACS) in the guinea pig: both BCV and ACS cause a stimulus-locked increase in spike discharge rate in the irregular utricular afferent neuron. (Modified from [13])

These experiments revealed that 500 Hz sound and vibration selectively activate type I vestibular hair cells in the utricular *and* saccular striola and their postsynaptic vestibular afferents with irregular resting discharge (Fig. 4). The combination of type I vestibular hair cells, ultrafast calyx synapses, and irregular otolith afferents is ideally suited for a stimulus- and phase-locked coding of these dynamic vestibular stimuli (500 changes in acceleration per second) with high temporal precision (see above and S1, S2 [online supplementary material] for more details).

In contrast, the resting activity of semicircular canal (both regular and irregular) and regular otolith afferents is virtually not altered during application of 500 Hz ACS or BCV at stimulus levels usually employed for clinical VEMP testing. Most probably, the sound- and vibration-induced endolymph flow in the semicircular canals is too weak to deflect hair cell stereocilia in the canal crista as long as the bony labyrinth is intact [75]. This may however change when a third mobile window is introduced into the bony wall of the inner ear in addition to the round and oval windows (see section “Superior canal dehiscence” and S4 [online supplemental material] for details).

## Neuronal projections

If sound and vibration are able to activate *both* utricular and saccular afferents, how can ocular and cervical VEMPs differentiate between utricular and saccular function? The predominant specificity of oVEMPs for utricular and cVEMPs for saccular function is not due to different stimulus qualities (i.e., ACS and BCV), but to the differential neuronal projections of utricular and saccular afferents in the central vestibular system.

Anatomical and neurophysiological evidence shows that utricular afferents project predominantly to vestibulo-ocular neurons in the vestibular hindbrain, while saccular afferents mainly contact vestibulo-spinal neurons [32, 40, 59, 77–79]. Therefore, ocular VEMPs recorded from the inferior oblique eye muscle predominantly reflect utricular function, and cervical VEMPs obtained from the SCM are predominantly an indicator of saccular function (Fig. 1a–c; [14, 17]).

At this point, it is important to notice that the separation between the central projections of utricular and saccular afferents is predominant, and not complete. Furthermore, 10% of the saccular afferents travel in the superior vestibular nerve together with the utricular afferents (see above and Fig. 2d). A number of clinical studies in patients with superior and inferior vestibular neuritis indicate, however, that the predominant specificity of oVEMPs for utricular and cVEMPs for saccular function is sufficient for distinguishing between utricular and saccular dysfunction in clinical practice (see also section “Vestibular neuritis” and [6, 11]).

To make things even more complicated, not only otolith, but also semicircular canal afferents project to vestibulo-ocular and vestibulo-spinal neurons in the vestibular hindbrain. For instance, afferents of the superior semicircular canal provide excitatory input to the contralateral inferior oblique muscle and inhibitory input to the ipsilateral SCM via central vestibular neurons [78, 79]. These pathways are however silent in subjects with a normally encased bony labyrinth, as the fluid displacement in the superior semicircular canal during application of 500 Hz sound and vibration is too small to activate the respective vestibular hair cells and afferents (see above). The “silent” projections may, however, become activated if a third mobile window is introduced into the bony labyrinth, e.g., in superior canal dehiscence, resulting in increased VEMP responses (see below and S4 [online supplemental material]).

## Measurement set-up and analysis of data

This section summarizes the most important principles concerning measurement set-up and data analysis of VEMP recordings. For more details, please refer to [26, 63, 74].

### Stimulus quality

Short ACS and BCV stimuli can be used for recording both o‑ and cVEMPs (for detailed stimulus parameters see below and [63, 68, 74]). A stimulus frequency of 500 Hz is generally recommended for most clinical applications because most of the neurophysiological data in the guinea pig model were obtained for 500 Hz sound or vibration [21, 74]. Please note that stimulus frequencies <500 Hz are able to activate irregular semicircular canal in addition to irregular otolith afferents—even in the normally encased bony labyrinth. As this might compromise the otolithic specificity of VEMPs, these frequencies are not recommended for VEMP testing in clinical practice [19].

Beside sound and vibration, galvanic currents applied to the mastoid via large surface electrodes can be employed as VEMP stimuli (galvanic or gVEMPs). So far, this technique has mainly been applied in experimental settings, and not in clinical routine [28].

**Fig. 5:**
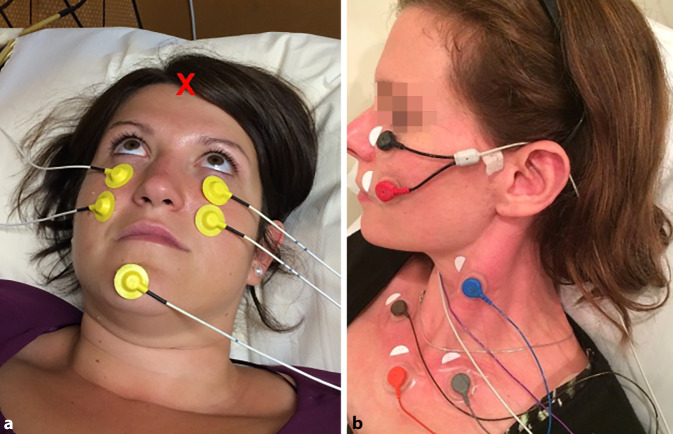
Placement of surface electrodes for the recording of **a** ocular and **b** cervical vestibular evoked myogenic potentials. *X:* Fz (midline of the forehead at the hairline)

*Air-conducted sound*: ACS (clicks or tone bursts) is presented to both ears sequentially with calibrated headphones or insert phones, while the ipsilateral cVEMP or the contralateral oVEMP response is recorded (Fig. 5). The test subject has to be protected from excessive sound exposure during presentation of ACS stimuli. When using ACS, the test subject has to be protected from excessive sound exposure.

When using ACS as a VEMP stimulus, the test subject has to be protected from excessive sound exposure

To achieve this aim, a careful calibration of the sound source is of paramount importance. As a rule of thumb, a sound pressure level (SPL) of 130 dB(A) applied for 1 s is regarded to be safe. The peak (p) SPL should not exceed 140 dB(A). Detailed information concerning the calculation of sound exposure and prevention of noise trauma has been provided by [7, 8, 68, 74]. If the sound pressure level is indicated in “dB nHL” (normal hearing level) by the manufacturer of the VEMP platform, it has to be converted into “dB pSPL” or “pe (peak equivalent) SPL” in order to calculate safe sound exposure levels. The individual conversion factor depends on multiple variables, such as headphone type, stimulus parameters (see below), and ear canal volume [39, 68]. Conversion factors for the most common test conditions are often provided in the manufacturer’s instructions (e.g., [38]), otherwise they have to be determined during calibration of the sound source.

The use of ACS stimuli requires functional integrity of the middle ear.

As a rule of thumb, a 500 Hz ACS tone burst of 95–100 dB nHL is required to obtain stable cVEMPs in a healthy subject, while 5–10 dB more are needed for oVEMPs, reflecting the higher sensitivity of irregular saccular as compared to utricular afferents to ACS in the guinea pig model [18, 72, 74].

Finally, the neurotologist should always be aware that the use of ACS stimuli requires intact middle ear function. An air–bone gap as small as 10 dB is able to absorb so much sound energy in the middle ear that the remaining energy finally reaching the labyrinth might be too small to evoke reliable VEMP responses [86].

The use of ACS stimuli requires functional integrity of the middle ear

*Bone-conducted vibration*: BCV is usually applied to the midline of the forehead at the hairline (Fz; Fig. 5a), from where the vibration propagates through the skull and causes simultaneous linear acceleration in both labyrinths with approximately equal intensity. Therefore, a time-saving simultaneous recording of right- and left-sided VEMP responses is possible in this set-up [42]. Furthermore, BCV does not put the test subject at risk of excessive sound exposure, as irregular otolith afferents are much more sensitive to BCV (thresholds of ~0.02 g) than ACS (thresholds >80 dB SPL) [21]. Vibration can also be applied in patients with conductive hearing loss, as the sound energy is transported to the labyrinth via bone and connective tissue, and not through the middle ear.

A conventional bone-conduction device (e.g., RadioEar B71) is usually too weak for application at Fz; therefore, more powerful electromechanical vibrators are recommended (e.g., minishaker type 4810, Bruel and Kjaer) along with an appropriate amplifier (e.g., power amplifier type 2718, Bruel and Kjaer) [41, 42]. It should however be noted that the latter are not certified medical devices; therefore, their use is currently restricted to scientific applications. Tapping Fz with a standard reflex hammer (which *is* a medical device) is a cost-effective alternative for delivering BCV to Fz in clinical practice (for technical details see [37, 41, 49]).

### Stimulus parameters

The amplitude of a VEMP response is not determined by the total duration of the stimulus, but by its abrupt start (“jerk”; [3, 48]). The latter is the adequate stimulus for activating the transient channel of otolithic function (see above). Therefore, short stimuli (up to 6 ms) with short rise times (0–2 ms) are generally recommended for evoking o‑ and cVEMPs in clinical practice. In general, 50–200 stimuli at a repetition rate of 5 pulses per second (pps) are required to obtain robust VEMP responses [74]. When using the reflex hammer, 10 taps to Fz are usually sufficient to evoke a stable signal, as this is an ideal “jerk” with a rise time of 0 ms [17, 37].

### Placement of recording electrodes

For recording of oVEMPs, the active electrode is placed on the skin covering the inferior oblique muscle, i.e., on the infraorbital rim in one line with the pupil, the reference electrode is located 1–2 cm further below (Fig. 5; [42]). The patient is asked to look upwards (“look at your forehead”, Fig. 5a) during the recording as this increases the relatively small oVEMP n10 amplitude (5–10 µV). This effect is caused by a combination of bringing the belly of the inferior oblique muscle closer to the recording electrode on the skin and increasing the tonic activity of the muscle during upward gaze [73].

The active electrode for recording cVEMPs is placed on the skin above the mid-third of the anterior arm of the SCM, the reference electrode is located either on the medial end of the clavicula (Fig. 5b) or on the sternum (in case only one reference electrode is used for the two sides). The ground electrode for both o‑ and cVEMPs can be fixed to the chin or sternum. A certain baseline activity of the SCM is required for recording cVEMPs, as these are inhibitory responses. To this end, the test subject is asked to lift their head straight ahead while lying in a semi-recumbent position on the examination table. If the baseline activity of the SCM is too low with this maneuver, the test subject is asked to lift the head and turn it to the contralateral side [74].

### Outcome parameters

Amplitudes and latencies of the oVEMP n10 and the cVEMP p13n23 responses are the most important outcome parameters in clinical practice. For a better comparison between right- and left-sided amplitudes, the asymmetry ratio (AR) is calculated:

$$\mathrm{AR}=|\text{right}-\text{left amplitude}|\div |\text{right}+\text{left amplitude}|\times 100 \%$$

An AR value >40% indicates an asymmetry between right- and left-sided dynamic otolithic function [42, 43, 86].

The “raw” cVEMP p13n23 amplitude (in µV) correlates linearly with the baseline activity of the SCM (in µV) [5]. Therefore, it is recommended to calculate a dimensionless “corrected” p13n23 amplitude: the raw amplitude (obtained from the unrectified averaged trace of the electromyograhic [EMG] recording) is divided by the baseline activity of the SCM. The latter is determined as the average amplitude of the rectified or root mean square (RMS) EMG recorded in parallel to the unrectified EMG in the interval between −20 ms and stimulus onset (0 ms). For details, see Figs. 5 and 6 in [74]. The corrected p13n23 amplitude allows for a better comparison of cVEMP responses recorded from different sides, in different individuals and during different recording sessions.

### Normal values

Normal values for VEMPs depend on many variables, such as the test subject’s age and various stimulus parameters (e.g., frequency, intensity, duration, rise times) [1, 3, 60, 67, 72, 85]. Therefore, it is strongly recommended that each neurotological center defines its own normal values for each set of stimulus parameters and different age groups [74].

## Clinical applications

The following section focuses on characteristic VEMP responses in peripheral vestibular disorders that have contributed to a better understanding of the neurophysiology behind VEMPs or that provide additional information as compared to other vestibular tests. Detailed accounts of clinical VEMP applications have been provided by, for example [14, 26, 46, 50, 57, 62, 70, 74, 81, 84].

### Vestibular neuritis

In line with the afferent innervation of the labyrinth (Fig. 2d), patients with a superior vestibular neuritis display reduced oVEMP n10 amplitudes on the contralateral side (Fig. 1e), while right- and left-sided cVEMPs are symmetrical. On the other hand, reduced ipsilateral cVEMP p13n23 amplitudes and symmetrical oVEMP n10 responses are a hallmark of inferior vestibular neuritis (Fig. 1f; [12, 33, 44, 51, 52, 56, 61, 64, 82]). This “double dissociation” of o‑ and c‑VEMPs in superior *versus* inferior vestibular neuritis has two important implications. First, it indicates that the two otolith-driven reflexes do not originate in the same subset of vestibular receptors. If they did, one would expect that either both responses or none at all would be affected in superior/inferior neuritis. Second, the double dissociation shows that the *predominant* specificity of oVEMPs for contralateral utricular function and cVEMPs for ipsilateral saccular function is sufficient to distinguish between utricular and saccular dysfunction in clinical practice—although the neuronal projections of utricular and saccular afferents are not completely separated [11].

### Otolith organ specific vestibular dysfunction

VEMPs are an efficient and reliable tool for detecting isolated disorders of the otolith organs [16, 30]. If vestibular testing assesses only semicircular canal function (e.g., by calorics or vHIT), these disorders may easily been missed and classified as “non-vestibular” or “functional”. Therefore, the results of semicircular canal testing have to be complemented by selective tests of otolithic function such as VEMPs in clinical practice.

VEMPs are an efficient and reliable tool for diagnosing otolith organ specific vestibular dysfunction

This is especially important in patients with mild traumatic brain injury or blast trauma who often suffer from isolated dysfunction of the otolith organs despite intact semicircular canal function [2, 47].

### Superior canal dehiscence

A dehiscence in the bony roof of the superior semicircular canal creates a third mobile window in the bony labyrinth (beside the oval and round windows) resulting, e.g., in sound-induced vertigo and nystagmus (Tullio phenomenon; for details see Infobox 1​ and S4 [online supplementary material]; [35, 83]).

**Fig. 6:**
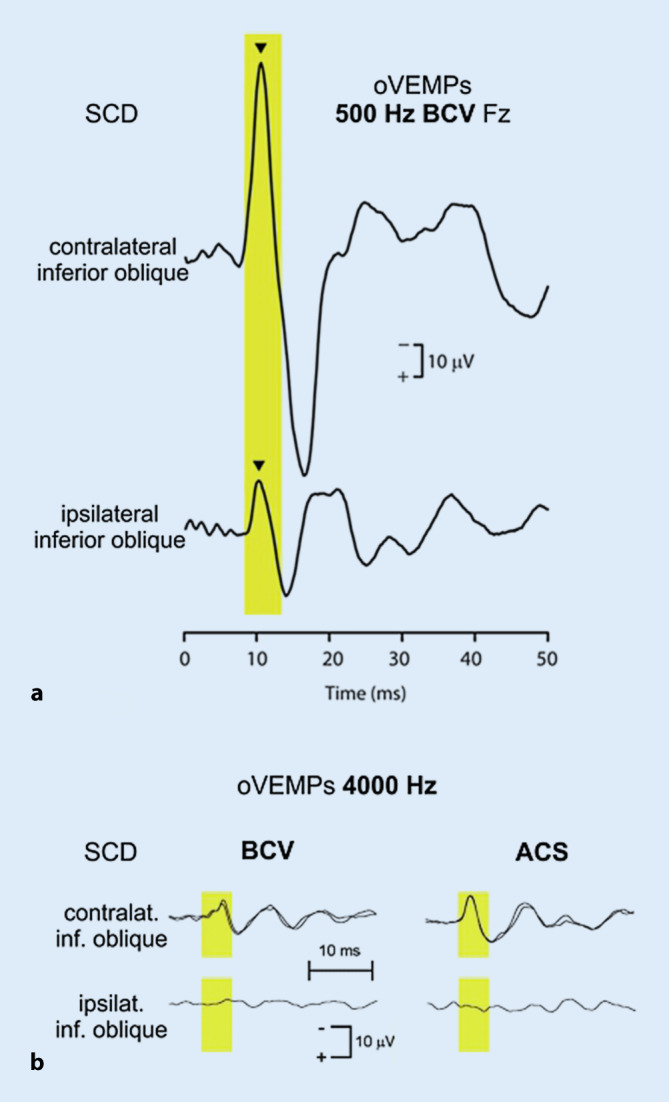
Effect of a superior canal dehiscence (*SCD*) on ocular vestibular evoked myogenic potentials (*oVEMPs*). **a** Enhanced contralateral oVEMP n10 amplitude (*arrowhead*) during application of 500 Hz bone-conducted vibration (*BCV*) at Fz (midline of the forehead at the hairline). (Reprinted from [54] with permission from © Wolters Kluwer Health, Inc.). **b** Presence of a contralateral oVEMP n10 response to 4000 Hz BCV and air-conducted sound (*ACS*) in a case of SCD. (Slightly modified and reprinted from [55] with permission from © SAGE Publications)

A highly increased oVEMP n10 response recorded from the inferior oblique muscle is a powerful indicator for a superior canal dehiscence (SCD) in the contralateral labyrinth with a sensitivity and specificity >90% depending on stimulus parameters and control populations (Fig. 6a; [45, 54, 80, 88]). Furthermore, patients with an SCD display a contralateral oVEMP n10 response to 4000 Hz ACS or BCV, which is absent in normal subjects with an intact bony labyrinth (Fig. 6b; diagnostic accuracy = 100%). As the latter test does not depend on comparison between right- and left-sided responses, it is ideally suited to diagnose bilateral SCD [55].

The tremendous effect of SCD on the oVEMP n10 amplitude can be explained by activation of the otherwise “silent” vestibulo-ocular projections of superior canal afferents (see section “Neuronal projections”). While semicircular canal hair cells and afferents do not respond to sound or vibration in the normally encased labyrinth, the third mobile window in SCD induces an endolymph flow in the superior semicircular canal [75] finally resulting in activation of type I vestibular hair cells and their postsynaptic irregular afferents. As these project to vestibulo-ocular neurons—just like utricular afferents—they increase the oVEMP n10 amplitude recorded from the inferior oblique eye muscle [4, 27, 79].

Following closure of the superior canal dehiscence in a guinea pig model of SCD, superior semicircular canal afferents do no longer respond to ACS and BCV, which is in line with the normalization of VEMP responses after successful surgical closure of SCD in humans [27, 87].

### The “difficult” patient

Most neurotological tests, such as caloric irrigation and vHIT, infer vestibular (dys‑)function from the video-oculographic (VOG) assessment of the vestibulo-ocular reflex, or put simply: you have to look into your patients’ eyes to determine their balance function. This may, however, be difficult or even impossible in certain groups of patients, e.g., those with congenital/infantile nystagmus (superimposition of the VOG recording by the infantile nystagmus), extremely poor vision (blind nystagmus; inability to fixate a target during vHIT), or reduced ability to cooperate (e.g., blink and lid artifacts). In these cases, o‑ and cVEMPs offer a simple, quick, and reliable alternative to obtain some basic information about superior (oVEMPs) and inferior vestibular nerve (cVEMPs) function (Fig. 2d; [14]). Both responses can be recorded in patients with infantile nystagmus [53]. Due to the short duration of the examination (40 s for a trial with 200 stimuli at 5 pps), VEMPs are also a suitable diagnostic tool for patients with limited ability to cooperate, e.g., children and patients with cognitive impairment.

#### Infobox 1 Background information

Websites accessed on 23 April 2020

- mode of function of a seismometer: https://www.iris.edu/hq/inclass/animation/seismograph_horizontal
- animation of the Tullio phenomenon:
  https://www.youtube.com/watch?v=UohkAL7IY0w

## Practical conclusion

- VEMPs (vestibular evoked myogenic potentials) are a simple, safe, reliable, and selective test of transient (= dynamic) otolithic function.
- 500 Hz ACS (air-conducted sound) and BCV (bone-conducted vibration) selectively activate type I vestibular hair cells and irregular otolith afferents in the utricular and saccular striola. The predominant specificity of oVEMPs (ocular vestibular evoked myogenic potentials) for contralateral utricular function and of cVEMPs (cervical vestibular evoked myogenic potentials) for ipsilateral saccular function is due to the differential projections of utricular and saccular afferents to vestibulo-ocular and vestibulo-spinal neurons, respectively.
- When using ACS, the test subject has to be protected from excessive sound exposure.
- The “raw” cVEMP p13n23 amplitude is corrected for the baseline activity of the sternocleidomastoid muscle determined by the rectified or root mean square EMG electromyogram for a better comparison between cVEMP responses from different recordings and test subjects.
- A highly increased oVEMP n10 amplitude is a diagnostic indicator for a contralateral superior canal dehiscence.
- Informative VEMP recordings can be obtained from patients with congenital/infantile nystagmus, extremely poor vision or limited ability to cooperate during vestibular testing.

## Caption Electronic Supplementary Material

Further information on the following topics: S1: The striola: an ideal “jerk” detector. S2: Sound and vibration as otolithic stimuli. S3: Vestibular microphonics. S4: Superior canal dehiscence

## Abbreviations

ACS: Air-conducted sound
AR: Asymmetry ratio
BCV: Bone-conducted vibration
c: Cervical
Fz: Midline of the forehead at the hairline
g: Gravitational acceleration
nHL: Normal hearing level
o: Ocular
p: Peak
pe: Peak equivalent
pps: Pulses per second
RMS: Root mean square
SCD: Superior canal dehiscence
SCM: Sternocleidomastoid muscle
SPL: Sound pressure level
VEMPs: Vestibular evoked myogenic potentials
vHIT: Video head impulse test
VOG: Video-oculography
