# Supplementary material for: Evidence-based diagnostic use of VEMPs: From neurophysiological principles to clinical application
Source: HNO. 2020 Jun 12;68(Suppl 2):69–78. doi: 10.1007/s00106-019-00767-2 (PMC7403168; doi:10.1007/s00106-019-00767-2)
Supplement: Supplementary file 1 — Further information on the following topics: S1: The striola: an ideal “jerk” detector. S2: Sound and vibration as otolithic stimuli. S3: Vestibular microphonics. S4: Superior canal dehiscence [file 106_2019_767_MOESM1_ESM.pdf]

Online supplementary material

„Evidence-based diagnostic use of VEMPs. From neurophysiological principles to clinical application“

Julia Dlugaiczyk

S1: The striola - an ideal „jerk“ detector

Vestibular hair cells and afferents in the striola of the otolith organs display several anatomical, biomechanical and neurophysiological pecularities as compared to the extrastriola, which make this region ideally suited for the detection of high-frequency changes in linear acceleration („jerks“) within the transient (=dynamic) channel of otolithic function (see [21, 23, 26, 58]).

First, the particularly short and stiff hair bundles of striolar hair cells are only tenuously attached to the overlying gel layer of the otoconial membrane and move freely within vertical endolymph-filled channels of the gel layer. Due to their stiffness, the stereocilia are deflected even by small shearing forces caused by relative motion between the neuro-epithelium and the otoconial layer. Furthermore, the hair bundles are almost instantenously dragged with the endolymph motion as they are only loosely attached to the gel layer above them. Thus, sound waves up to 3 kHz (i.e. 3,000 cycles of fluid displacement per second) are translated into almost synchronous oscillations of the receptor potential in striolar vestibular hair cells (for details see S3 and [65, 66]).

The biomechanical properties of striolar hair cells are complemented by the high temporal precision of the ultra-fast cup-shaped calyx synapses between type I striolar hair cells and their postsynaptic afferents (Fig. 2b, c; [76]). The highly specialized synaptic cleft is able to maintain high concentrations of K^+ ions that depolarize the hair cell membrane (thus increasing the likelihood of transmitter release) and the postsynaptic vestibular afferent (thus bringing it closer

to the threshold for triggering action potentials) [9]. This so called „non-quantal“ synaptic transmission (in addition to the „quantal“ excytosis of glutamate vesicles) enhances the temporal precision of the calyx synapse required to encode high-frequency changes in linear acceleration [22, 23].

Finally, striolar hair cells are mainly contacted by vestibular afferents with irregular resting discharge that are particularly sensitive to transient (=dynamic) vestibular stimuli due to their membrane properties [31]. In summary, all these specializations of the striola form the basis for encoding fast changes in linear acclereration with high temporal precision. This is the prerequisite for using sound and vibration with stimulus frequencies of 500 Hz and more as stimuli for VEMP recordings (see S2 and [22, 23]).

S2: Sound and vibration as otolithic stimuli

Going back to physics, a sound wave (ACS or BCV) is defined as an oscillation of pressure propagated through a medium, i.e. a repetitive acceleration and deceleration of particles within the medium. This principle is illustrated by an animation of a longitudinal sound wave (https://www.docsity.com/en/news/physics/physics-sound-visual-representation-gifs/) and the sound-induced periodic movement of styrofoam beads in a Kundt’s tube (https://www.youtube.com/watch?v=NHAHR5ingRU) (websites accessed on April 23rd, 2020).

Accordingly, each cycle of an ACS or BCV stimulus is able to evoke a pressure wave within the vestibulum resulting in periodic movements of the utricular and saccular maculae and subsequent deflection of their hair cell stereocilia. A stimulus frequency of 500 Hz is equivalent to 500 changes in acceleration per second – an ideal jerk stimulus [21, 26].

S3: Vestibular microphonics

The effect of ACS and BCV on vestibular hair cells of the utricular macula was analyzed in detail in a guinea pig model: oscillations of the utricular macula evoked by application of sound or vibration to the guinea pig's skull were measured by laser Doppler vibrometry showing that the utricular macula moved with the stimulus frequency up to several kHz. Simultaneous recordings of utricular microphonics revealed a periodic de- and repolarisation of the utricular hair cells, again in sync with the stimulus frequency [65, 66].

In summary, these experiments provide evidence that sound and vibration are able to induce motion in the utricular macula with subsequent hair cell de- and repolarisation, which is the prerequisite for activation of otolith afferents during VEMP recordings.

S4: Superior canal dehiscence

In superior canal dehiscence (SCD), a defect in the thin bone of the middle cranial fossa overlying the superior (= anterior) semicircular canal creates a third mobile window in the labyrinth – beside the round and oval windows. If sound energy enters the inner ear *via* the oval window in SCD, part of the energy is shunted away from the cochlea through the newly created low-impedance pathway in the superior semicircular canal towards the third window. The resulting endolymph displacement is now strong enough to deflect the hair cell stereocilia in the crista of the superior canal – quite in contrast to the situation in the intact bony labyrinth – resulting in the typical clinical signs and symptoms of SCD, such as sound-induced nystagmus and vertigo (Tullio phenomenon; see Infobox and [35, 75, 83]).

SCD is frequently overdiagnosed in high-resolution computed tomography (HRCT) of the temporal bone with 1 mm slice thickness. Therefore, a resolution of ≤ 0.6 mm in HRCT is

recommended for the diagnosis of SCD in addition to at least one clinical symptom and one audio-vestibular finding compatible with a third window, e.g. increased VEMP amplitudes of the affected labyrinth [83]. Ocular VEMPs provide a higher diagnostic accuracy for detecting SCD than cVEMPs [88].
